# Deciphering of differences in gut microbiota and plasma metabolites profile between non-obese and obese Golden Retrievers dogs

**DOI:** 10.3389/fmicb.2024.1514633

**Published:** 2025-01-08

**Authors:** Yazhen Cai, Huayu Tang, Guilin Xiang, Hongyu Yi, Jie Zhong, Zhaoxi Xie, Qinfeng Hu, Romaissaa El Bouhi, Pan Zhou, Yong Zhang, Honglin Yan

**Affiliations:** School of Life Science and Engineering, Southwest University of Science and Technology, Mianyang, China

**Keywords:** obesity, Golden Retriever, serum biochemistry indexes, plasma metabolome, microbiota

## Abstract

**Introduction:**

Golden Retrievers have a high risk of obesity, which is prevalent in dogs and is associated with inflammation and cancer, impairing the health and life expectancy of companion animals. Microbial and metabolite biomarkers have been proposed for identifying the presence of obesity in humans and rodents. However, the effects of obesity on the microbiome and metabolome of Golden Retrievers remains unknown. Therefore, this study was designed to evaluate the signatures of serum biochemistry indexes, gut microbiota and plasma metabolites in non-obese and obese Golden Retrievers, aiming to recognize potential biomarkers of canine obesity.

**Methods:**

A total of 8 non-obese (Ctrl group) and 8 obese (Obe group) Golden Retrievers were included in the present study to collect blood and feces samples for measurements. The fecal microbiome and plasma metabolome were determined using 16S rRNA amplicon sequencing and liquid chromatography-mass spectrometry, respectively.

**Results:**

Results showed that the alanine aminotransferase activity and total bilirubin concentration, which have been measured using serum biochemistry analysis, were higher in the Obe group than in the Ctrl group (*p* < 0.05). Moreover, there was a significant difference in gut microbiota composition between the two groups (*p* < 0.05). The phyla Proteobacteria, Fusobacteriota, and Bacteroidota as well as genera *Fusobacterium*, *Prevotella*, *Faecalibacterium*, *Escherichia*-*Shigell*, and *Alloprevotella* were more abundant, while phylum Firmicutes and genera *Peptoclostridium*, *Blautia*, *Turicibacter*, *Allobaculum*, and *Erysipelatoclostridium* were less abundant in the Obe group compared to the Ctrl group (*p* < 0.05). Plasma concentrations of citrulline and 11-dehydrocorticosterone were significantly higher in the Obe group than those in the Ctrl group (*p* < 0.05). Close correlations between serum biochemistry parameters, gut microbiome, and plasma metabolites were observed in the current study.

**Conclusion:**

The obesity-induced shifts in serum biochemistry indexes, gut microbiota, and plasma metabolites profiles suggest that obese Golden Retrievers exhibit a different microbiome and metabolome than non-obese ones, and the certain metabolites like citrulline and 11-dehydrocorticosterone could be considered as potential biomarkers to recognize obese Golden Retrievers.

## 1 Introduction

Obesity has been declared as a major global health problem in both human beings and domesticated animals, and it has been most commonly observed in companion animals (Chandler et al., 2017). Previous studies have showed that approximately 59% of dogs are overweight or obese (Courcier et al., 2010). Obese dogs have been demonstrated to be more prone to suffering from a subclinical inflammatory state than their lean counterparts, characterized by the higher levels of certain inflammatory markers, which are associated with a higher risk of cardiovascular illnesses and cancer (Vecchiato et al., 2023). Thus, accurate diagnosis is crucial for early obesity treatment in dogs, thereby enhancing their welfare and quality of life. The most common tool used by veterinarians to assess the nutritional status of dogs is the body condition score (BCS) system (Broome et al., 2023), which has relatively high accuracy and reproducibility, but it still has a certain degree of subjectivity in determining canine obesity because the evaluation primarily relies on visual examination and palpation (Tesi et al., 2020; Miller et al., 2018). Even among experienced veterinarians, discrepancies in evaluating BCS for the same dog may occur. Therefore, the additional biomarker indicators are needed to identify for providing supplementary references in the diagnosis of canine obesity by the veterinarians using BCS system.

Serum biochemistry parameters are commonly used to detect the metabolic health of humans and animals. Alanine aminotransferase (ALT) and total bilirubin (TB) serve as significant potential biomarkers in the clinical assessment of liver inflammation-related diseases not only in humans, but also in feline and canine populations (Dirksen et al., 2017; Kozat and Sepehrizadeh, 2017). Furthermore, recent research has demonstrated a positive correlation between ALT levels and body weight in humans, highlighting its potential utility in evaluating metabolic health (Bekkelund and Jorde, 2019).

Although obesity is highly prevalent in dogs, there are higher frequencies of overweight conditions in certain breeds of dogs including Golden Retrievers, Pug, Beagle and English Springer Spanie than in others (Reddy et al., 2019; Pegram et al., 2021). The breed susceptibility of Golden Retriever to obesity is 49.53% according to the clinical study (Mounika et al., 2020), implying that Golden Retrievers should be paid more attentions on their obese status (Pegram et al., 2021; O’Neill et al., 2021). In recent years, increasing evidence has shown that gut microbiota dysbiosis played a causal role in the early onset of obesity and lipid metabolism dysfunction (Yu et al., 2019; Zhang and Gérard, 2022). On the other hand, obese individuals have harbored a distinct gut microbiome from those lean individuals, indicated by reduced microbial diversity and altered taxonomic abundances in the overweight cohort (Lippert et al., 2017). The higher Firmicutes and Bacteroidetes ratio has been related to obesity in some studies, but these observations are inconsistent across studies, and it is unknown which specific microbial taxon is directly related to the development of obesity (Xie et al., 2012). Likewise, overweight Beagle dogs also have exhibited a significantly different gut microbiome from the normal ones, characterized by higher abundances of genera *Faecalibacterium*, *Phascolarctobacterium*, *Megamonas*, *Bacteroides*, *Mucispirillum* (Kim et al., 2023). These evidences further support the contention that gut microbiota may be involved in the metabolic health of the host. Owing to the close relationship between gut microbiota and host obesity, several attempts have been made to reveal the potential microbial biomarkers for detecting dietary responsiveness in obese individuals with impaired metabolic health. Indeed, some microbial biomarkers have been identified to hold the potential to predict obesity, and interventions based on these microbial biomarkers might be beneficial to weight loss and metabolic risk improvement (Barengolts et al., 2019). It remains unknown the differences in gut microbiota composition between non-obese and obese Golden Retriever dogs, and thus it is necessary to identify microbial biomarkers of obesity in Golden Retrievers.

Gut microbiota dysbiosis in obese individuals has been shown to affect host physiology possibly via modulating the metabolism and excretion of microbial-derived metabolites including lipids and lipid-like metabolites, amino acids, bile acids derivatives, and catabolites of plant bioactive components, which were also proposed to be predictors of overweight or obesity (Machate et al., 2020; Lippert et al., 2017). Metabolomics has been enlightened as a useful tool to evaluate changes in metabolites due to overweight and obesity at the cellular level and body fluid level (Xie et al., 2012). Thus, metabolite profiling with metabolomics might represent an opportunity to methodically establish biomarkers for obesity diagnosis and control that are relatively simple to measure in comparison to traditional approaches like the BCS system (Rauschert et al., 2014).

Therefore, the alterations in composition of the gut microbiota and the plasma metabolites profile may be informative in predicting the obese status of Golden Retrievers. The present study adopted microbiome and metabolomics techniques to test the differences in gut microbiota and plasma metabolites profile between non-obese and obese Golden Retrievers to reveal the potential obesity biomarkers from microbiota and/or metabolites.

## 2 Materials and methods

The experimental protocols related to animal treatment in the present study were approved by the Institutional Animal Care and Use Committee of Southwest University of Science and Technology (No. L2023029).

### 2.1 Animals and management

**Figure 1:**
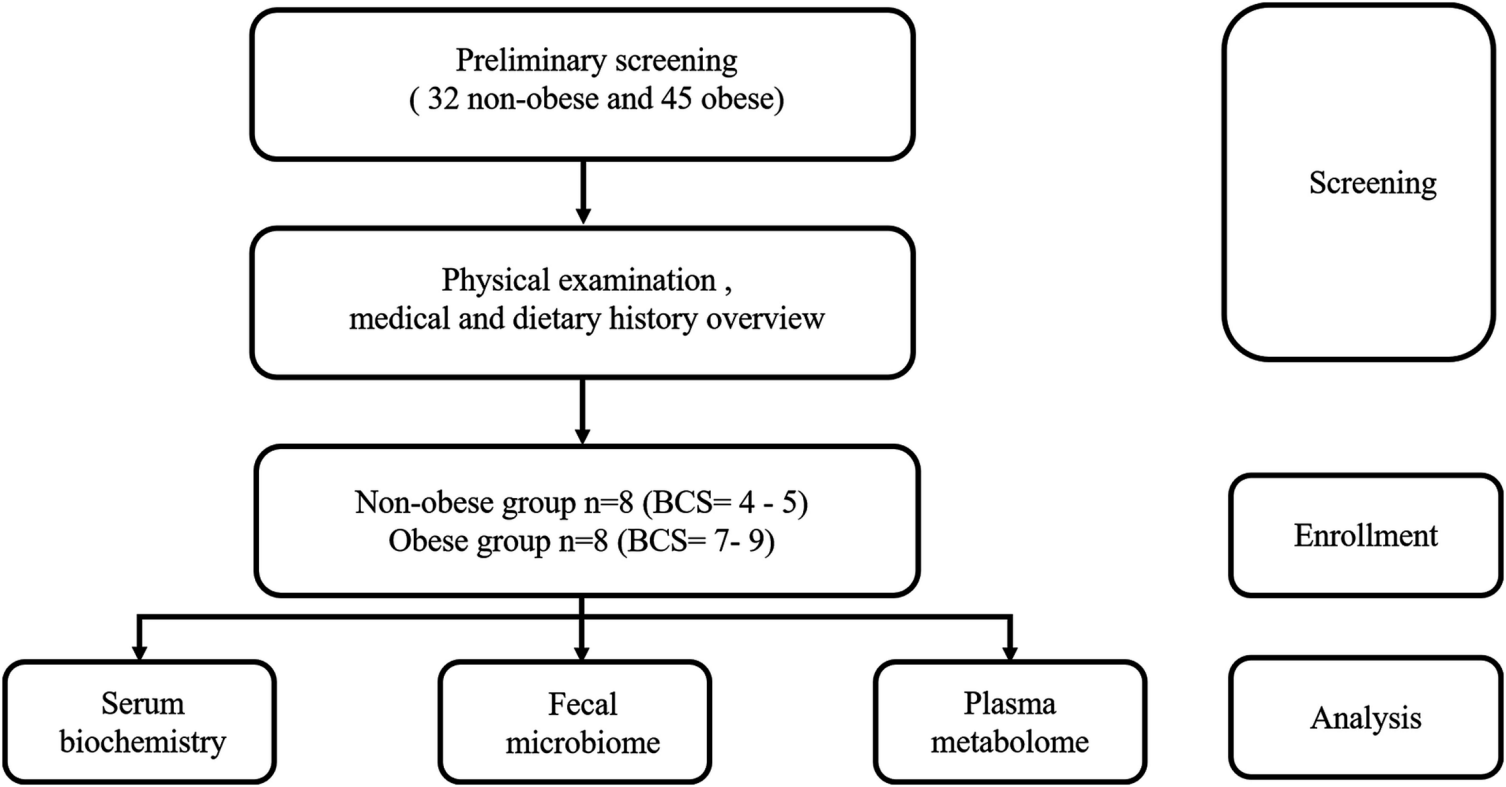
Flow diagram of study design.

The design of the analytical workflow is shown in Figure 1, which illustrates the enrollment of groups. Before screening, a total of 77 Golden Retrievers applicants were registered by the owners to participate in the study. Dogs were excluded if they had a history of drugs or antibiotics administration within a month. Importantly, dietary history was also considered as a crucial screening factor. Only the Golden Retrievers consuming diets with similar nutrient levels and ingredients composition over 3 months were kept for the further screening procedures. All feeds were formulated primarily with duck and chicken meat. In addition, the physical examinations of all Golden Retrievers were performed to exclude those with obvious inflammatory disease and to assess the BCS according to a validated 9-point BCS system. The dogs with a BCS range from 4 to 5 were the non-obese objects in the present study, with a BCS range from 7 to 9 defined as the obese studying objects and their counterparts (Broome et al., 2023). Accordingly, those with BCS at other scores were excluded as well. After screening, 8 non-obese (Ctrl group) and 8 obese (Obe group) adult Golden Retrievers (Males = 11; Females = 5) aged between 1 and 7 years were enrolled in this study. The average body weight of the Ctrl and Obe groups (n = 8) were 27.00 ± 9.16 kg and 39.00 ± 10.38 kg, and the average BCS of the Ctrl and Obe groups were 4.9 and 7.2, respectively (Table 1). The dietary history and obesity duration details of these 16 Golden Retrievers were listed in Supplementary Table 1, and the nutrient levels of diets for the dogs were presented in Supplementary Table 2.

**Table 1:** Characteristics of non-obese (Ctrl) and obese (Obe) Golden Retriever dogs included in the present study.

Group	Name	Gender	Age	BW (kg)	BCS (V1)	BCS (V2)	BCS (Ave)	Diet	Calories intake (Kcal)
Ctrl	Dada	Female	1.00	25.00	5.00	5.00	5.00	Diet 4	1686.00
	Fugui	Female	5.50	25.00	4.00	4.00	4.00	Diet 3	1777.00
	Zhaozhao	Female	1.00	23.00	5.00	5.00	5.00	Diet 4	1686.00
	Legou	Male	6.50	29.00	5.00	5.00	5.00	Diet 2	1764.00
	Pipi	Male	1.00	23.00	5.00	4.00	4.50	Diet 1	1715.00
	Niangao	Male	2.00	33.00	5.00	5.00	5.00	Diet 2	1764.00
	Jiuyi	Female	1.00	30.00	5.00	5.00	5.00	Diet 1	1715.00
	Xilin	Male	2.00	35.00	5.00	5.00	5.00	Diet 1	1715.00
Obe	Mocha	Male	5.00	40.00	7.00	7.00	7.00	Diet 1	1715.00
	Dingman	Male	4.00	35.50	7.00	7.00	7.00	Diet 3	1777.00
	Pangguo	Male	3.00	40.00	8.00	7.00	7.50	Diet 3	1777.00
	Rouwan	Male	5.00	47.00	8.00	8.00	8.00	Diet 4	1686.00
	Hanzai	Male	3.00	35.00	7.00	7.00	7.00	Diet 1	1715.00
	Otto	Male	2.00	44.00	8.00	7.00	7.50	Diet 2	1764.00
	Nicol	Female	2.00	33.00	7.00	7.00	7.00	Diet 2	1764.00
	Guolicheng	Male	3.50	33.00	7.00	7.00	7.00	Diet 2	1764.00

Ctrl, control group, of which the body condition score (BCS) ranges from 4 to 5; Obe obese group, of which the BCS ranges from 7 to 9; BCS (V1) (V2), body condition score were determined by the first veterinarian and the second veterinarian respectively; BCS (Ave), the average body condition scores of two veterinarians BW, body weight.

### 2.2 Sample collection

Spontaneous excreted fecal samples were collected by the owner following the procedures under the guidance of veterinarians. After the fecal samples were delivered to the laboratory, the samples were kept at −20°C until DNA extraction and the following analysis. Blood samples were collected by the veterinarians from the forelimb veins into vials with or without anticoagulants. After collection, plasma and serum samples were obtained by centrifuging the blood at 2,500 × *g* for 15 min. Plasma and serum aliquots were stored at −20°C pending metabolomics analysis and biochemical index measurement, respectively. All of the collection processes were conducted under the supervision of the veterinarians.

### 2.3 Serum biochemistry analysis

The concentrations of glucose, creatinine, blood urea nitrogen, phosphorus, calcium, total protein, albumin, globulin, TB, and cholesterol, along with the activities of ALT, alkaline phosphatase, glutamyl transferase, amylase, and lipase, were analyzed using a veterinary automatic biochemical analyzer (IDEXX Procyte Dx, IDEXX Laboratories, USA) by the colorimetric method previously used for canine blood biochemistry. Subsequently, the blood urea nitrogen/creatinine ratio and the albumin/globulin ratio were calculated.

### 2.4 Fecal analysis

Genomic DNA in feces samples of dogs were extracted using the TIANamp Soil DNA Kit DP336 (TIANGE, China) according to the manufacturer’s instructions. The quantity and integrity of isolated DNA were determined on a NanoDrop ND-1000 instrument and evaluated visually by agarose gel electrophoresis, respectively. The V4 region of the bacterial 16S rRNA gene was amplified using 515F (GTGCCAGCMGCCGCGGTAA) and 806R (GGACTACHVGGGTWTCTAAT). Sequencing libraries were generated and indexes were added. The 16S rRNA amplicon sequencing was performed by the company (Novogene, China) on the Illumina MiSeq platform through NovaSeq6000 PE25. Software FLASH (Version 1.2.11) and Fastp (Version 0.23.1) were used to process the raw data to exclude low-quality reads. The resulting clean reads were assembled into effective tags, which were assigned to the Operational taxonomic unit (OTUs) based on 97% similarity by Uparse (Version 7.0. 1,001). Taxonomic classification of OTU clusters was performed using the Greengenes database with RDP (Version 2.6). The alpha diversity indexes were calculated by R studio (Version 4.1) accordingly. The Bray-Curtis distance was used to compare the structural difference of the microbiota communities across samples and was visualized by principal coordinates analysis (PCoA) to demonstrate the clustering of different samples using a vegan package in R studio (Version 3.4.1). The raw sequencing data can be obtained by searching against the National Center for Biotechnology Information databases with the project number PRJNA1158542.

### 2.5 Plasma metabolomics analysis

Plasma samples were thawed at room temperature before analysis. 100 μL of samples were placed into polyethylene tubes, mixed with 400 μL of 50% methanol buffer, then vortexed for 30 s, and sonicated for 10 min in a 4°C water bath. After that, the samples were vortexed (1 min) and centrifuged (14,000 g, 15 min, 4°C) to collect the supernatant for nontargeted metabolite profiling. LC–MS/MS analysis was performed on an ultra-high performance liquid chromatography system (Vanquish, Thermo Fisher Scientific) coupled to an Orbitrap Exploris 120. To monitor the stability of the analysis, the QC sample was obtained by pooling all the experimental samples with equal volume. The raw data were processed through ProteoWizard and BiotreeDB (Version 3.0). The metabolites were identified by comparing with the internal database mass-to-charge ratio (m/z), retention time, and chromatographic data. The partial least-square discriminant analysis (PLS-DA) was performed using the MetaboAnalyst 6.0 web-based system. The metabolites with *p* < 0.05 were considered significantly different. The significantly distinct metabolites were imported into the MetaboAnalyst 6.0 database for the pathway enrichment analysis.

### 2.6 Statistical methods

The body condition indexes and serum biochemistry parameters were analyzed using the student *t*-test with SAS software (Version 9.4). The non-parametric Wilcoxon rank-sum test was adopted to analyze the differences in abundances of microbial taxa at the phylum and genus levels as well as the alpha diversity indices. The Spearman’s correlations between the predominant genera and plasma-differed metabolites or serum biochemistry parameters as well as the correlation between plasma- differed metabolites or serum biochemistry indexes were calculated and demonstrated as heatmaps by R studio (Version 4.0.3). The Benjamini–Hochberg method was applied to calculate the false discovery rates (FDR) adjusted *p*-value. Data were expressed as mean ± SEM, and significance was declared at *p* < 0.05.

## 3 Results

### 3.1 Body condition and serum biochemistry indexes

The results related to body condition and serum biochemistry parameters are listed in Table 2. A significant difference in body weight and BCS was observed between the Ctrl and Obe groups (*p* < 0.05). There was a tendency toward higher activity of ALT (*p* = 0.09) in the serum of obese dogs compared to their non-obese counterparts. A higher concentration of total bilirubin was found in the Obe group than that in the Ctrl group (*p* < 0.05). Moreover, there were no significant differences in other indicators between the two groups (*p* > 0.05).

**Table 2:** The differences in body condition and serum biochemistry parameters between the Ctrl and Obe group.

Parameters	Ctrl	Obe	*p*-value
Body Weight (kg)	27.88 ± 1.62	38.44 ± 1.84	<0.001
Body Condition Score	4.81 ± 0.13	7.25 ± 0.13	<0.001
Glucose (mg/dL)	80.88 ± 7.00	84.38 ± 6.28	0.715
Creatinine (mg/dL)	1.04 ± 0.02	1.09 ± 0.05	0.426
Blood urea nitrogen (mg/dL)	17.5 ± 1.46	17.25 ± 1.25	0.899
Blood urea nitrogen/Creatinine	17.13 ± 1.25	15.29 ± 1.10	0.287
Phosphorus (mg/dL)	3.66 ± 0.23	3.56 ± 0.24	0.754
Calcium (mg/dL)	9.74 ± 0.13	9.54 ± 0.10	0.256
Total protein (g/dL)	6.71 ± 0.16	6.64 ± 0.08	0.688
Albumin (g/dL)	3.31 ± 0.03	3.42 ± 0.07	0.218
Globulin (g/dL)	3.36 ± 0.16	3.18 ± 0.05	0.273
Albumin/Globulin (%)	1.00 ± 0.05	1.10 ± 0.05	0.152
Alanine aminotransferase (U/L)	30.75 ± 2.07	39.88 ± 4.57	0.090
Alkaline phosphatase (U/L)	50.00 ± 7.00	44.38 ± 4.84	0.519
Glutamyl transferase (U/L)	0.01 ± 0.01	0.50 ± 0.38	0.302
Total bilirubin (mg/dL)	0.29 ± 0.02	0.35 ± 0.02	0.011
Cholesterol (mg/dL)	219.63 ± 11.02	220.25 ± 19.83	0.210
Amylase (U/L)	745.63 ± 101.6	721.38 ± 82.15	0.855
Lipase (U/L)	448.25 ± 56.98	598.25 ± 75.72	0.136

Ctrl, control group, of which the body condition score (BCS) ranges from 4 to 5; Obe obese group, of which the BCS ranges from 7 to 9.

### 3.2 Gut microbiota composition

An average of 99,790 high-quality sequences were obtained from all samples, with a range of 97,361 to 107,162. These sequences were assigned to 265 OTU as the core OTU. Among them, 157 OTU were found in both groups, while 35 and 73 OTU were found in Ctrl and Obe groups, respectively (Supplementary Figure 2).

**Figure 2:**
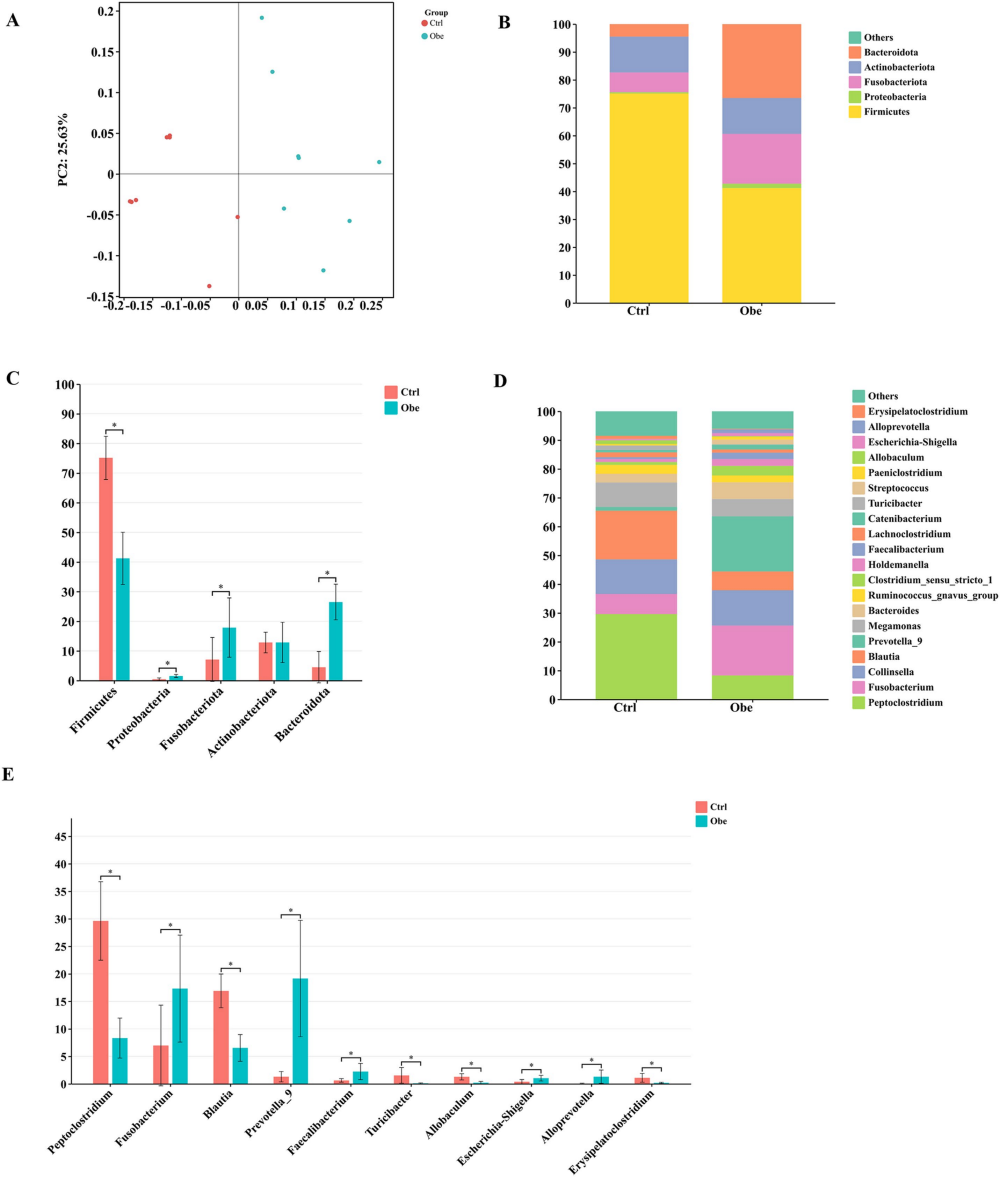
The beta diversity and taxonomic distribution of fecal microbiota in the Ctrl and Obe dogs. **(A)** principal coordinates analysis (PCoA) plot of unweighted UniFrac distances of fecal microbial communities. **(B)** The stacked plot of dominant taxa at the phylum level. **(C)** Significantly different phyla between the Ctrl and Obe groups. **(D)** The stacked plot of dominant taxa at the genus level. **(E)** Significantly different genera between the Ctrl and Obe groups.

No significant differences were detected in *α* diversity indices (Supplementary Figure 1) between Ctrl and Obe groups. The PCoA plot showed that the Ctrl and Obe samples could form two distinct clusters by treatment (Figure 2A). This indicates that the composition of the microbiota of the Ctrl and Obe groups was different.

At the phylum level, Firmicutes, Proteobacteria, Fusobacteriota, Actinobacteriota, and Bacteroidota were predominant taxa in the feces of dogs. Moreover, the presence of phylum Firmicutes was the most dominant taxon in both groups, accounting for 75.12 and 41.21% of the fecal microbiota in the Ctrl and Obe groups, respectively (Figure 2B; Supplementary Table 3). Besides, Proteobacteria, Fusobacteriota, and Bactercidota were found to be more abundant in the feces of the Obe group compared to the Ctrl group (*p* < 0.05) (Figure 2C; Supplementary Table 3). Firmicutes/Bacteroidota ratio were 1.55 and 16.73%, respectively, in Obe and Ctrl group. At the genus level, the most predominant taxon in feces of the Ctrl and Obe groups was *Peptoelostridium* and *Prevotella*, respectively (Figure 2D; Supplementary Table 4). Compared to the Ctrl group, the abundances of Fusobacterium, *Prevotella*, *Faecalibacterium*, *Escherichia*-*Shigell* and *Alloprevotella* were higher and the abundances of *Peptoclostridium*, *Blautia*, *Turicibacter*, *Allobaculum*, and *Erysipelatoclostridium* were lower in the feces of Obe group (*p* < 0.05) (Figure 2E; Supplementary Table 4).

### 3.3 Plasma metabolic pathways of non-obese and obese Golden Retrievers

**Figure 3:**
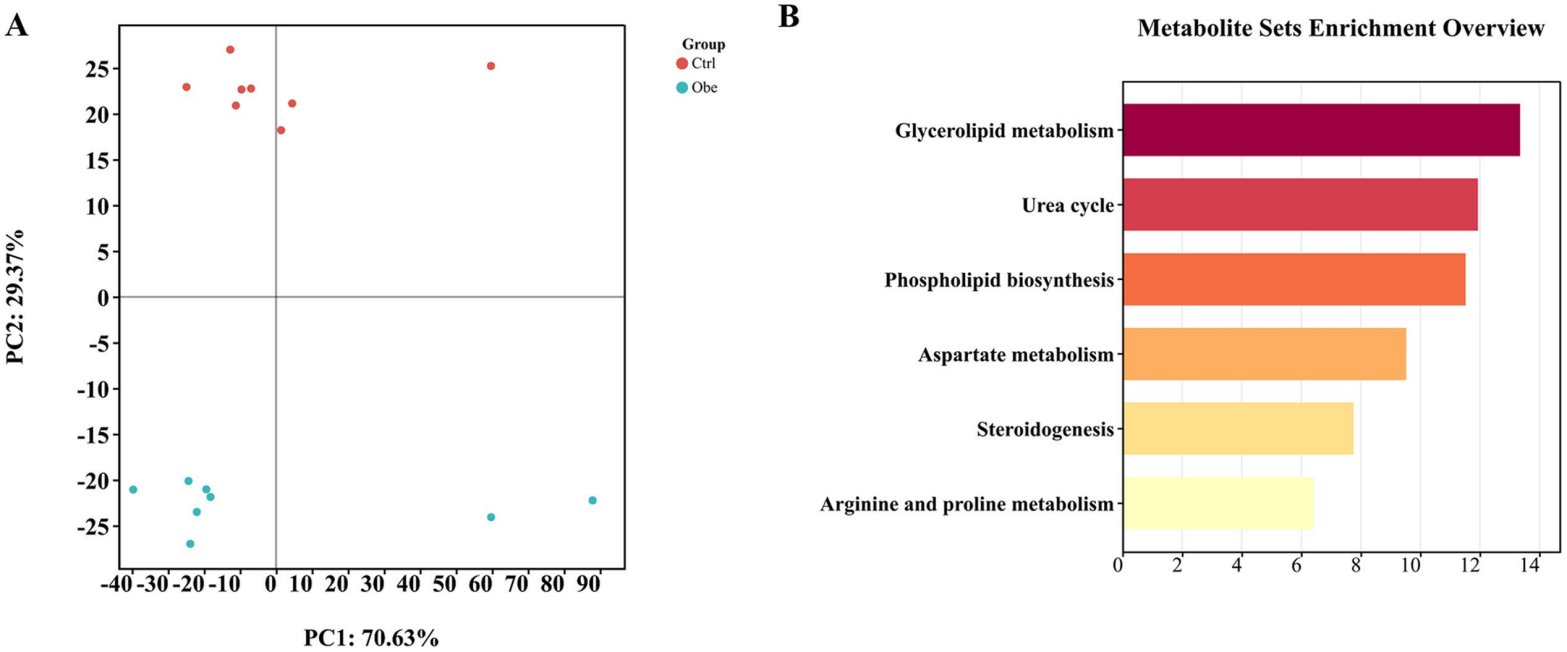
Plasma metabolomics profile. **(A)** Partial least-square discriminant analysis plot based on plasma metabolites. **(B)** Metabolic pathway enrichment analysis.

The PLS-DA score plot (Supplementary Figure 4) from the mass spectrometer data showed that the distribution of the quality control samples was clustered tightly, demonstrating the accuracy of the method for metabolomics data acquisition. In the PLS-DA plot (Figure 3A), Ctrl and Obe samples were clustered separately, indicating that there was a remarkable difference in plasma metabolomics profile between the Ctrl and Obe groups.

A total of 10 significantly different metabolites were identified (Table 3). Among them, citrulline, PA (16:0/16:0), 3-Methoxy-4-Hydroxyphenylglycol sulfate, 11-dehydrocorticosterone, ditryptophenaline and N-alpha-Acetyl-L-citrulline were found to be more abundant and N-hydroxy-L-isoeucine, pelargonic acid and 8-iso-15-keto-PGE2 were less abundant in the Obe group compared to the Ctrl group (*p* < 0.05).

**Table 3:** Significantly different metabolites in plasma between the Ctrl and Obe group.

Parameters	Ctrl	Obe	*p*-value
Citrulline	0.11 ± 0.01	0.14 ± 0.01	0.038
N-Hydroxy-L-isoleucine	2.06 ± 0.29	1.18 ± 0.18	0.021
Pelargonic acid	2.61 ± 0.14	1.86 ± 0.18	0.006
PA (16:0/16:0)	0.15 ± 0.02	0.25 ± 0.03	0.018
3-Methoxy-4-Hydroxyphenylglycol sulfate	0.79 ± 0.10	1.24 ± 0.15	0.022
11-Dehydrocorticosterone	21.8 ± 3.45	35.97 ± 3.43	0.011
8-is0-15-keto-PGE2	0.21 ± 0.03	0.10 ± 0.02	0.006
Canrenone	12.57 ± 2.00	20.00 ± 2.19	0.025
Ditryptophenaline	19.48 ± 2.87	34.05 ± 3.79	0.008
N-alpha-Acetyl-L-citrulline	0.13 ± 0.01	0.18 ± 0.02	0.045

Ctrl, control group, of which the body condition score (BCS) ranges from 4 to 5; Obe obese group, of which the BCS ranges from 7 to 9.

The different metabolites were checked against the SMPDB database to match the corresponding metabolic pathways and the biological pathways (Figure 3B). The differently enriched metabolites were mainly involved in glycerolipid metabolism, urea cycle, phospholipid biosynthesis, steroidogenesis, aspartate metabolism as well as arginine and proline metabolism.

### 3.4 The correlation between fecal microbiota, plasma metabolites and serum biochemistry index

**Figure 4:**
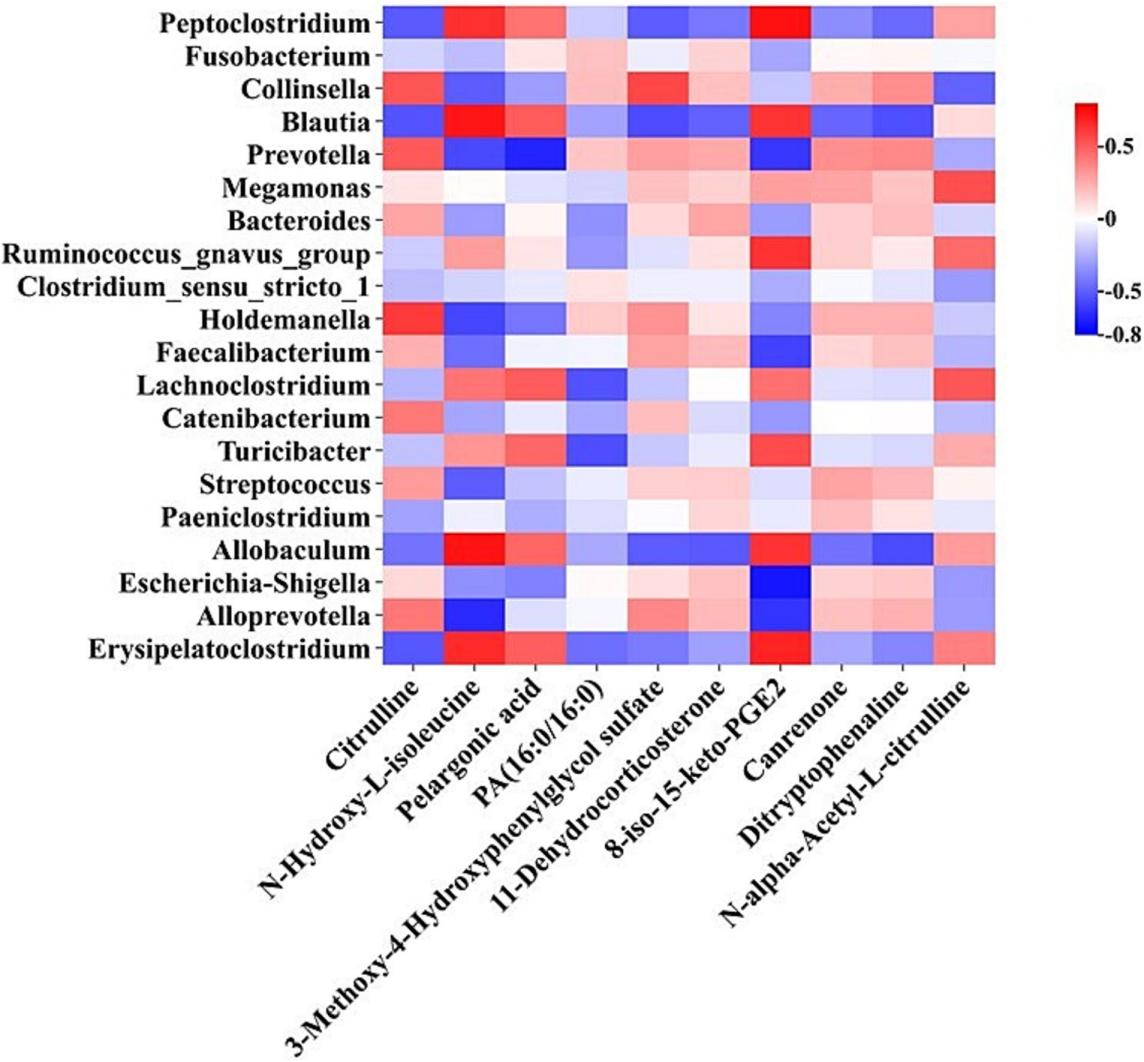
The correlation analysis between the altered genera and plasma distinct metabolites.

The correlation analyses between the altered genera and plasma distinct metabolites, which were identified by 16S rRNA sequencing and LC–MS-based untargeted metabolomics, respectively, were performed and visualized in a heat map (Figure 4). The levels of genera *Peptoclostridium*, *Blautia*, *Allobaculum*, and *Erysipelatoclostridium* were positively related to plasma concentrations of 8-iso-15-keto-PGE2 and N-Hydroxy-L-isoleucine (*p* < 0.05), while the abundances of genera *Prevotella* and *Alloprevotella* were negatively related to plasma levels of these two metabolites (*p* < 0.05). The abundance of genus Escherichia-Shigella was negatively associated with the plasma content of 8-iso-15-keto-PGE2 (*p* < 0.05). Besides, the levels of genera *Prevotella* and *Collinsella* were in positive connection with the plasma citrulline level, while the abundance of genus *Blautia* was in negative connection with its level in plasma (*p* < 0.05).

The correlation analyses between plasma metabolites and serum biochemistry indexes as well as the fecal microbiota and serum biochemistry parameters were performed and visualized in heat maps. Plasma concentrations of metabolites 8-iso-15-keto-PGE2 and N-alpha-Acetyl-L-citrulline were negatively related to aminotransferase activity (*p* < 0.05). The content of metabolite 3-Methoxy-4-Hydroxyphenylglycol sulfate was in a positive relation with serum TB level (*p* < 0.05) (Supplementary Figure 3A). Furthermore, the abundances of genera *Escherichia*-*Shigella*, *Clostridium sensu stricto* 1, and *Catenibacterium* were positively related to serum aminotransferase activity (*p* < 0.05). The abundances of genera *Allobaculum* and *Faecalibacterium* were in a negative and positive relation with the serum concentration of TB, respectively (*p* < 0.05) (Supplementary Figure 3B).

## 4 Discussion

Obesity has reached pandemic proportions in dogs (Courcier et al., 2010; Butterwick, 2000), in which has significant implications for canine physiology, health and welfare, especially causing gut microbiota dysbiosis and metabolism dysfunction. Previous research has identified potential microbial and metabolomic biomarkers associated with obesity in humans and rodents (Metwaly et al., 2022; Wang et al., 2021; Giudetti, 2023). However, the similar kinds of biomarkers for diagnosis of canine obesity remain unclear. Accordingly, the present study compares the serum biochemical indices, fecal microbiota and plasma metabolites profile of non-obese and obese Golden Retrievers to preliminarily reveal the potential microbial or metabolic biomarkers for obesity in Golden Retrievers.

Studies in both humans and animals have showed that obesity causes the disruption of the homeostasis of lipid metabolism and increases fat accumulation not only in adipose tissue but also in the liver, resulting in adipose inflammation and hepatic damage (Giudetti, 2023; Richard et al., 2000). Obesity-associated fatty liver has been shown to induce a substantial increase in the activity of ALT, which suggests a potential connection between ALT and obesity. A previous study has demonstrated that obese dogs exhibited higher activities of ALT and gamma-glutamyl transferase (Tribuddharatana et al., 2011). Consistently, a tendency toward higher activity of ALT was found in the obese Golden Retrievers compared to the control dogs. Besides, previous study found that serum TB was associated with an elevated risk of obesity-associated metabolic syndrome (Wei et al., 2021). TB including direct and indirect bilirubin, the degradation products of the heme moiety of hemoglobin and other hemoproteins, is potentially toxic to the liver where it is detoxified (Roy-Chowdhury et al., 2020). Bilirubin has been proposed as a biomarker to monitor hepatic function and of which the high level in blood was the consequences of liver dysfunction according to the previous study (Guerra Ruiz et al., 2021). In the present study, a significantly higher level of bilirubin was found in the Obe group, indicating the potential liver impairment in overweight dogs.

Gut microbiota play a crucial role in host metabolism and physiology, but the pathophysiological mechanisms and clinical outcomes were still unclear. On one hand, the presence of obesity has been associated with the imbalance of gut microbiota, which has been demonstrated to play a causal role in the induction of metabolic dysfunction (Le Chatelier et al., 2013; Stanislawski et al., 2019). On the other hand, several studies observed significant differences in the abundance and biodiversity of gut microbiota between non-obese and obese individual (Pinart et al., 2021; Yun et al., 2017). The Firmicutes/Bacteroidota ratio was considered as a microbial indicator for obesity by some studies, as the higher abundance of phylum Firmicutes and increased ratio of Firmicutes/Bacteroidota were observed in obese individuals or animals than their lean counterparts. However, others hold the different opinion that there is a weak connection between obesity and the Firmicutes/ Bacteroidota ratio (Schwiertz et al., 2010). Previous studies showed that the most predominant taxa at the phylum level in dogs were Firmicutes (You and Kim, 2021), which is also the most abundant phylum in the feces of Golden Retrievers. In addition, phylum Firmicutes has been found more abundant in non-obese dogs than the obese ones (Macedo et al., 2022; Park et al., 2015; Kim et al., 2023). Consistent with these findings, non-obese Golden Retrievers exhibited the higher level of phylum Firmicutes in feces than the Obe group, implying that phylum Firmicutes might be negatively correlated with the presence of obesity in dogs, which needs to be further verified in the future study. Genus *Fusobacterium* was found related to the whole-body inflammation state and has been more abundant in the fecal microbiota of children with obesity as well as in the feces of type 2 diabetes subjects (Sedighi et al., 2017; Yuan et al., 2021; Chen et al., 2020; Lee et al., 2018). In agreement with these findings, the increased abundance of genus *Fusobacteria* was observed in the Obe group in the present study. A previous study showed that fat pigs had significantly higher proportions of *Prevotella*. *copri* (Chen et al., 2021). However, a higher abundance of genus *Prevotella* has been associated with enhanced insulin sensitivity in rodents. These contradictory results might stem from the inter-study difference in the genetic background of studying animals, which has been proved as a determinant factor for the microbiota composition. A previous study on dogs showed that the abundance of *Prevotella*. *copri* in fecal microbiota elevated with increasing BCS (You and Kim, 2021). Consistently, in the present study, obese Golden Retrievers exhibited higher levels of genus *Prevotella* in feces. Likewise, another study also demonstrated that high carbohydrate diet-fed obese dogs exhibited higher abundance of *Prevotella*. *copri* in feces than dogs with normal weight (Li et al., 2017). Previous study compared the microbiota differences between dogs with differed degree of overweight have found that highly overweighed dogs had higher abundance of *Prevotella* in feces compared to their counterparts (Forster et al., 2018). Therefore, the abundance of *Prevotella* might be used as a potential biomarker for the detection of obesity in Golden Retrievers, but its effects on and reasons for its abundance in obese dogs should be examined in the future study. A higher level of *Peptoclostridium* was found in dogs with normal BCS compared to those with high BCS in a previous study on Beagle dogs (Kim et al., 2023). Consistently, in this study, the obese Golden Retrievers exhibited a lower abundance of *Peptoclostridium* in feces. Genus *Blautia* has been reported to exhibit beneficial effects on intestinal health and metabolic disorders (Liu et al., 2021). A previous study showed that oral administration of *Blautia* can ameliorates obesity and type 2 diabetes by increasing the production of short chain fatty acids (SCFAs) and activating SCFAs-related signaling pathways (Patel and Preedy, 2017). In the present study, non-obese Golden Retrievers had higher levels of genus *Blautia* in feces, implying that increasing the colonization of genus *Blautia* might be helpful for weight management in dogs, which needs to be studied in future research. The alterations in abundances of genus *Ruminococcus*_*gnavus*_*group* might the unique response of Golden Retrievers to obesity, because overweight had no effects on the abundances of these genera in other dog breeds like Beagle dogs (Kim et al., 2023). Therefore, the verification of the application of the genus as a biomarker indicator of obesity should be further studied.

The metabolite profiling via metabolomics is frequently used to reveal metabolic biomarkers for obesity and associated metabolic syndrome. In the present study, the plasma metabolite profile in obese Golden Retrievers differed from the non-obese dogs, which was consistent with previous findings that the presence of obesity significantly altered the whole-body metabolism in humans and rodents (Lai et al., 2014). Citrulline was produced by the hydrolysis of peptidyl arginine catalyzed by protein arginine deiminases, of which high activity was observed in a host of human diseases including metabolic syndrome and inflammation (Allerton et al., 2018). The conversion process of peptidyl arginine into citrulline also called protein citrullination, has been demonstrated to be an inflammation-dependent process since it is upregulated in various inflammatory states (Makrygiannakis et al., 2006). In this study, the plasma level of citrulline was higher in obese Golden Retrievers than in the control ones, suggesting that the presence of obesity might activate this posttranslational process, namely protein citrullination, in tissues of Golden Retrievers dogs. Besides, a previous study found that chronic administration of 11-dehydrocorticosterone to mice increased the circulating glucocorticoid level and downregulated the expression of genes related to the hypothalamic–pituitary–adrenal axis, leading to insulin resistance, adiposity and an elevation of body weight (Marzolla et al., 2014). In the current study, a higher concentration of 11-dehydrocorticosterone in plasma was observed in obese Golden Retrievers, indicating that the elevated cortisol secretion might contribute to the pathogenesis of obesity in Golden Retrievers (Abraham et al., 2013).

The catabolites of prostaglandin E2 (PGE2), including 15-keto-PGE2 and 8-iso-15-keto-PGE2, have been shown to alleviate hepatic inflammation in diet-induced obesity mouse model, indicative of lower activities of ALT and aspartate transferase and inhibited macrophage infiltration (Hee et al., 2023). Consistently, lower plasma levels of 8-iso-15-keto-PGE2 in obese dogs and the negative correlation between 8-iso-15-keto-PGE2 and aminotransferase activity were observed in the current study, suggesting that the approaches increasing the levels of catabolites of PGE2 might be effective for the treatment of obesity in Golden Retrievers. N-hydroxy-L-isoleucine is a derivative of L-isoleucine which has been shown to enhance muscle growth, inhibit fat deposition, and exhibit anti-inflammation activity (Patil et al., 2012). Given the beneficial effects of L-isoleucine on the metabolism and inflammation of the host, it can be speculated that N-hydroxy-L-isoleucine might exhibit similar effects as well. In the present study, obese dogs exhibited lower concentrations of N-hydroxy-L-isoleucine, implying that the intake of L-isoleucine and its derivatives might be useful for controlling overweight in dogs.

Overall, obesity significantly altered the concentrations of certain serum biochemistry parameters, which might be associated with the changes in gut microbiota and plasma metabolites profile. The verification of these possible biomarkers for diagnosing obesity in Golden Retrievers and the dietary intervention approaches targeting the microbiome and metabolome for weight management in dogs need to be further studied and explored.

## 5 Conclusion

In summary, obesity notably modified the levels of specific serum biochemical markers, which may correlate with alterations in gut microbiota and plasma metabolite profiles. Further investigation and exploration are necessary to validate these potential biomarkers for diagnosing obesity in Golden Retrievers, alongside dietary intervention strategies aimed at the microbiome and metabolome to manage weight in dogs.

## Data Availability

The datasets presented in this study can be found in online repositories. The names of the repository/repositories and accession number(s) can be found in the article/Supplementary material.
