# Supplementary material for: Deciphering of differences in gut microbiota and plasma metabolites profile between non-obese and obese Golden Retrievers dogs
Source: Front Microbiol. 2025 Jan 8;15:1514633. doi: 10.3389/fmicb.2024.1514633 (PMC11751222; doi:10.3389/fmicb.2024.1514633)
Supplement: Supplementary file 1 [file Supplementary_file_1.pdf]

Supplementary Material

1 Supplementary Figures and Tables

1.1 Supplementary Table

Supplementary Tabe 1. Dietary history and obesity duration of non-obese (Ctrl) and obese (Obe) Golden Retriever dogs included in the present study

Group	Name	Gender	Age (years)	Obesity duration (year)	Diet	Daily food intake (g)
Ctrl	Dada	Female	1.00	-	Diet 4	500
	Fugui	Female	5.50	-	Diet 3	500
	Zhaozhao	Female	1.00	-	Diet 4	500
	Legou	Male	6.50	-	Diet 2	500
	Pipi	Male	1.00	-	Diet 1	500
	Niangao	Male	2.00	-	Diet 2	500
	Jiuyi	Female	1.00	-	Diet 1	500
	Xilin	Male	2.00	-	Diet 1	500
Obe	Mocha	Male	5.00	Over 1 year	Diet 1	500
	Dingman	Male	4.00	Over 1 year	Diet 3	500
	Pangguo	Male	3.00	Over 1 year	Diet 3	500
	Rouwan	Male	5.00	Over 1 year	Diet 4	500
	Hanzai	Male	3.00	Over 1 year	Diet 1	500
	Otto	Male	2.00	Over 1 year	Diet 2	500
	Nicol	Female	2.00	Over 1 year	Diet 2	500
	Guolicheng	Male	3.50	Over 1 year	Diet 2	500

Ctrl, control group, of which the body condition score (BCS) ranges from 4 to 5; Obe obese group, of which the BCS ranges from 7 to 9;

Supplementary Tabe 2. Nutrient levels of commercial diets containing same protein ingredients

Nutrient levels	Diet 1	Diet 2	Diet 3	Diet 4
Metabolic energy, Kcal /kg	3430	3528	3554	3372
Crude protein (%)	28.00	27.00	26.00	28.00
Ether extract (%)	14.00	14.00	14.00	13.00
Ash (%)	10.00	10.00	8.00	10.00
Crude fiber (%)	5.00	5.00	4.00	5.00
Calcium (%)	1.00	1.20	1.20	1.00
Phosphorus (%)	0.80	1.00	0.80	0.80
Lysine (%)	1.00	1.20	1.00	1.20
Water soluble chloride (%)	0.30	0.45	0.45	0.45

Analyzed value provided by the companies；

Supplementary Tabe 3. The dominant phylum (average abundance > 1%) in the Ctrl and Obe group

Treatment	Phylum	Average abundance (%)
Ctrl	*Firmicutes*	75.12
	Fusobacteriota	7.09
	Actinobacteriota	12.83
	Bacteroidota	4.49
Obe	*Firmicutes*	41.21
	Proteobacteria	1.55
	Fusobacteriota	17.87
	Bacteroidota	26.49

Ctrl, control group, of which the body condition score (BCS) ranges from 4 to 5; Obe obese group, of which the BCS range from 7 to 9;

Supplementary Tabe 4. The dominant genus (average abundance > 1%) in the Ctrl and Obe group

Treatment	Genus	Average abundance (%)
Ctrl	*Peptoclostridium*	29.61
	Fusobacterium	6.98
	Collinsella	12.02
	Blautia	16.90
	Prevotella	1.30
	Megamonas	8.50
	Bacteroides	3.00
	Ruminococcus_gnavus_group	3.10
	Holdemanella	1.02
	Lachnoclostridium	1.69
	Turicibacter	1.54
	Allobaculum	1.20
	Erysipelatoclostridium	1.10
Obe	*Peptoclostridium*	8.32
	Fusobacterium	17.31
	Collinsella	12.24
	Blautia	6.53
	Prevotella	19.14
	Megamonas	5.98
	Bacteroides	5.85
	Ruminococcus_gnavus_group	2.33
	Clostridium_sensu_stricto_1	3.34
	Holdemanella	2.35
	Faecalibacterium	2.24
	Lachnoclostridium	1.11
	Catenibacterium	1.67
	Streptococcus	1.62
	Paeniclostridium	1.03
	Escherichia-Shigella	1.04
	Alloprevotella	1.30

Ctrl, control group, of which the body condition score (BCS) range from 4 to 5; Obe obese group, of which the BCS range from 7 to 9;

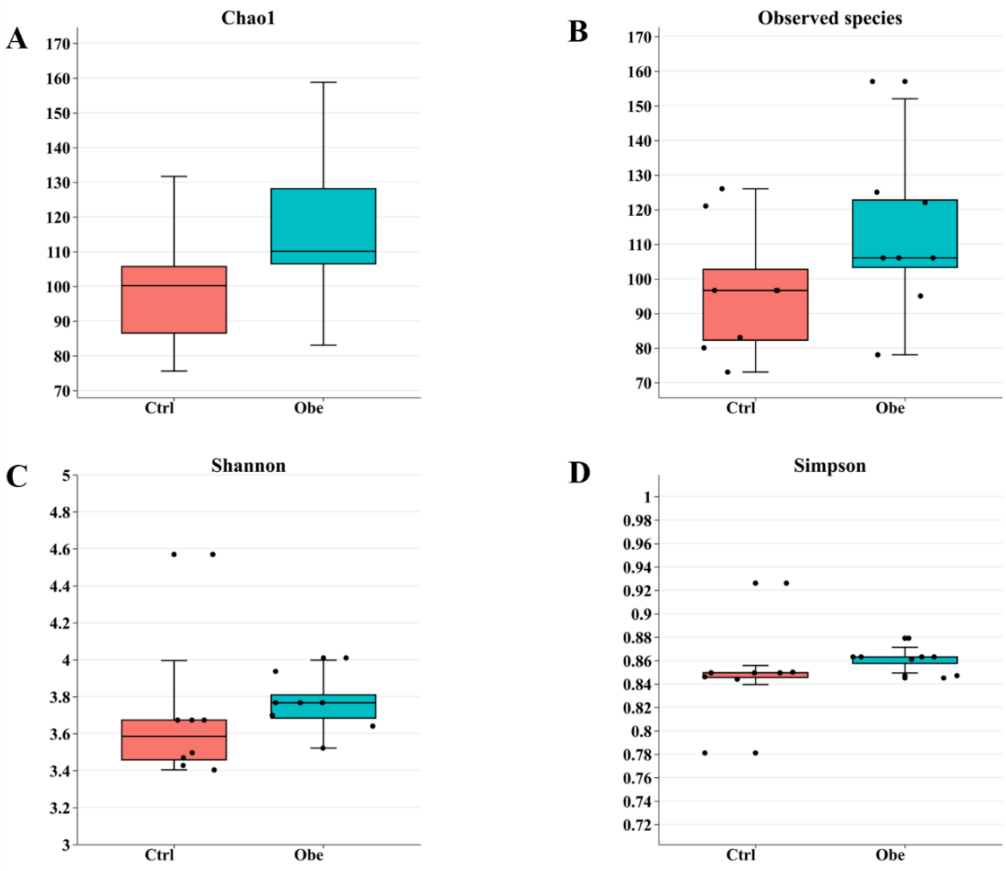

Supplementary Figure 1. The alpha diversity indices of fecal microbiota in the Ctrl and the Obe group. (A) Chao 1. (B) Observed species. (C) Shannon. (D)Simpson.

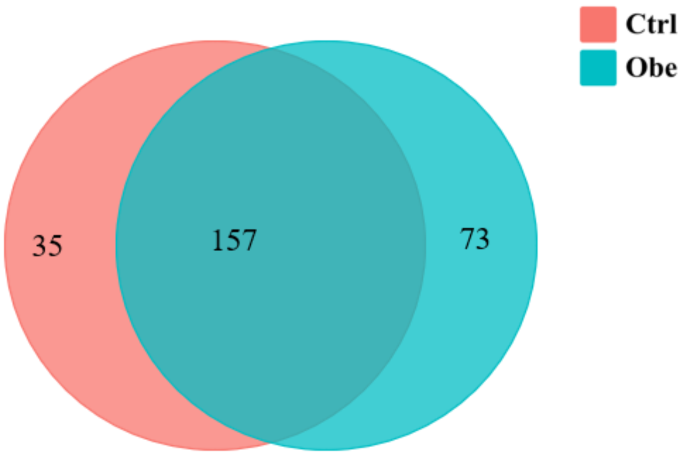

Supplementary Figure 2. The Veen analysis between Ctrl and Obe group.

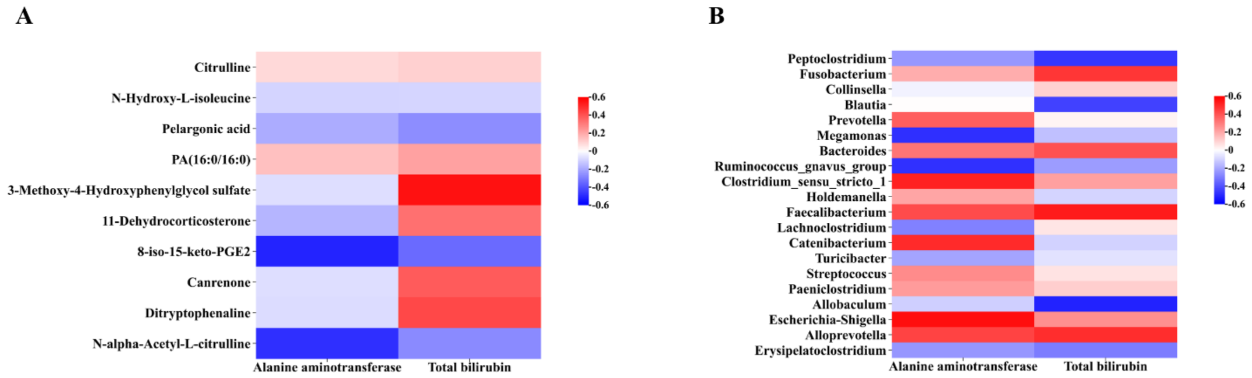

Supplementary Figure 3. The correlation analysis. (A) Plasma metabolites and serum biochemistry indexes. (B) fecal microbiota and serum biochemistry parameters.

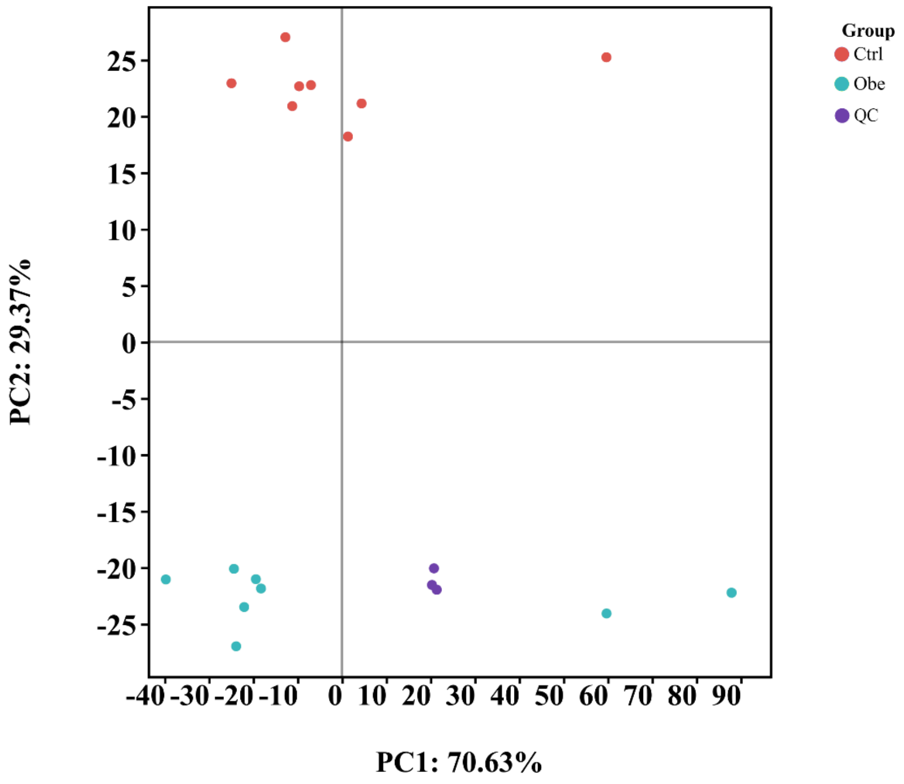

Supplementary Figure 4. The QC sample distribution in the PLS-DA diagram
